# Analysis of the Hippocampal Proteome in ME7 Prion Disease Reveals a Predominant Astrocytic Signature and Highlights the Brain-restricted Production of Clusterin in Chronic Neurodegeneration*

**DOI:** 10.1074/jbc.M113.502690

**Published:** 2013-12-23

**Authors:** Ayodeji A. Asuni, Bryony Gray, Joanne Bailey, Paul Skipp, V. Hugh Perry, Vincent O'Connor

**Affiliations:** From the ‡ Centre for Biological Sciences and; § Centre for Proteomic Research, University of Southampton, Southampton SO17 1BJ, United Kingdom

**Keywords:** Astrocytes, Mass Spectrometry (MS), mRNA, Neurobiology, Prions, ME7, Neurodegeneration, Prion, iTRAQ

## Abstract

Prion diseases are characterized by accumulation of misfolded protein, gliosis, synaptic dysfunction, and ultimately neuronal loss. This sequence, mirroring key features of Alzheimer disease, is modeled well in ME7 prion disease. We used iTRAQ^TM^/mass spectrometry to compare the hippocampal proteome in control and late-stage ME7 animals. The observed changes associated with reactive glia highlighted some specific proteins that dominate the proteome in late-stage disease. Four of the up-regulated proteins (GFAP, high affinity glutamate transporter (EAAT-2), apo-J (Clusterin), and peroxiredoxin-6) are selectively expressed in astrocytes, but astrocyte proliferation does not contribute to their up-regulation. The known functional role of these proteins suggests this response acts against protein misfolding, excitotoxicity, and neurotoxic reactive oxygen species. A recent convergence of genome-wide association studies and the peripheral measurement of circulating levels of acute phase proteins have focused attention on Clusterin as a modifier of late-stage Alzheimer disease and a biomarker for advanced neurodegeneration. Since ME7 animals allow independent measurement of acute phase proteins in the brain and circulation, we extended our investigation to address whether changes in the brain proteome are detectable in blood. We found no difference in the circulating levels of Clusterin in late-stage prion disease when animals will show behavioral decline, accumulation of misfolded protein, and dramatic synaptic and neuronal loss. This does not preclude an important role of Clusterin in late-stage disease, but it cautions against the assumption that brain levels provide a surrogate peripheral measure for the progression of brain degeneration.

## Teaser

**Background:** Prion disease causes chronic neurodegeneration via deposition of extracellular misfolded protein akin to Alzheimer disease.

**Results:** Proteomic analysis revealed a major nonproliferative astrogliosis encompassing GFAP, EAAT-2, Prdx6, and the acute phase protein Clusterin.

**Conclusion:** Brain changes are not reflective of measured circulating Clusterin.

**Significance:** Our data highlights caution in the use of circulating levels of biomarker as clinical measures of actual neurodegeneration.

## Introduction

Prion disease and Alzheimer disease (AD)^2^ are among a number of chronic neurodegenerative disorders in which the accumulation of misfolded protein is associated with neuropathology (1–3). In prion diseases, the generation of the misfolding insult can be sporadic, genetic, or of an infectious origin (4). Prion disease progression is characterized by activated microglia, astrogliosis, vacuolation, spongiform degeneration, and neuronal loss (5), and many of these neuropathological features are observed in AD (6). Central to this neuropathology is misfolding of plasma membrane-localized cellular prion protein (PrP^c^), leading to a predominantly extracellular accumulation of the conformationally altered isoform PrP^Sc^ (7–10). Although degeneration of neurons is associated with the accumulation of the misfolded protein, there is significant support for the idea that both intracellular and extracellular misfolded proteins play a pivotal role in the neuron loss in prion and other neurodegenerative diseases (11–13).

The degeneration of neurons in prion disease may be a cell autonomous event or involve interactions with other cells (14). In prion disease, there is evidence that expression of PrP^c^ in astrocytes alone is sufficient to support propagation of the disease progression and neurodegeneration (15). Besides being implicated in disease pathogenesis, astrocytes have been demonstrated to be neuroprotective (16). Typically, astrocytes contribute to a variety of functions in the brain, including homeostasis, neurotransmission, synapse formation, plasticity, and metabolism. In AD, the key components of the pathology are directly sensed by the glial populations that ordinarily support neuronal homeostasis leading to gliosis with poorly resolved functional consequences (17, 18).

In prion disease, the well characterized microglial signature shows that these cells are associated with an inflammatory response but one that is atypical and indicative of cells that may contribute to phagocytic clearance of debris (19, 20). Despite the anti-inflammatory profile associated with microglia in prion disease, inhibition of microglia proliferation even at the onset of disease symptoms can delay the emergence of behavioral deficits, reduce neurodegeneration, and prolong life (21). To provide a better understanding of the role of different cell types in prion disease progression, we carried out comparative hippocampal proteome analysis of control and ME7 animals at late-stage disease using isobaric tags for relative and absolute quantification (iTRAQ^TM^) and mass spectrometry. We profiled and quantified differences in the mRNA and protein expression of hippocampi from control and diseased animals at selected time points of disease progression and relate these to previously described cellular and behavioral dysfunction (22). This analysis suggests that the astrocytes dominate the proteome of diseased animals and mount a complex biochemical response. The response involves a range of activities that have the potential to ameliorate protein misfolding (apo-J and Clusterin), glutamate toxicity (EAAT-2), and oxidative stress (peroxiredoxin-6, Prdx6).

## EXPERIMENTAL PROCEDURES

### 

#### 

##### Stereotaxic Injection

These experiments were carried out in accordance with the United Kingdom Animals (Scientific Procedures) Act of 1986 and adhered to ARRIVE guidelines for reporting experiments involving animals (23). All surgical procedures were carried out as described previously (24). Briefly, age-matched female C57BL/6J mice were anesthetized and stereotaxically injected bilaterally into the hippocampus with 1 μl of 10% (w/v) brain homogenate prepared from normal brains (NBH) or from terminally ill ME7-infected mice. Onset of the clinical disease was measured weekly by determination of body weight. NBH- and ME7-infected mice were sacrificed at 8, 13, and 21 weeks post-injection to characterize early, middle, and late stages of disease. Mice were housed in a temperature- and humidity-controlled environment and had free access to food and water. Animals were terminally anesthetized and perfused with heparinized saline solution, and the micro-dissected hippocampus was frozen on dry ice.

##### iTRAQ Procedure

We pooled and homogenized hippocampi from NBH and ME7 animals (*n* = 5 per group) at 21 weeks post-injection in 10% w/v 0.5 m triethylammonium bicarbonate (iTRAQ kit, Applied Biosystems) containing 0.1% SDS and Complete protease inhibitors (Roche Applied Science). The protein concentration was estimated using protein assay from Bio-Rad and 100 μg from NBH and ME7 homogenates isotopically labeled using iTRAQ multiplex reagents (Applied Biosystems) as described by us and others (25, 26). The lysates were reduced with 2.5 mm tris(2-carboxyethyl)phosphine at 60 °C for 1 h and then alkylated using 10 mm methyl methane-thiosulfonate for 10 min at RT. These samples were proteolytically digested using trypsin at 37 °C for 20 h and lyophilized *in vacuo* before resuspension in 20 μl of 0.5 m triethylammonium bicarbonate. iTRAQ reagents 115 and 117 were resuspended in 70 μl of ethanol and added to the NBH and ME7 samples, respectively. This mixture was incubated at RT for 1 h before being quenched with 5 volumes of 0.1% TFA in water. Labeled samples were pooled and submitted to strong cation exchange fractionation on a Dionex Ultimate HPLC system (Dionex-LC Packings, Sunnyvale, CA) using a Phenomenex Luna, 5 μm, strong cation exchange column (150 × 4.6 mm, inner diameter). Forty fractions were collected at 200 μl/min and dried *in vacuo*. These fractions were reconstituted in 30 μl of 5% acetonitrile/water prior to nanoLC-MS/MS analysis.

##### NanoLC Tandem Mass Spectrometry

NanoLC-MSMS was performed using a CapLC system (Waters, Manchester, UK) coupled to a Streamselect micro-column switching module (Waters) on line to a Q-Tof Global Ultima (Waters). One-third of each sample was loaded via an autosampler onto a PepMap RP-C18 guard column (5 × 300 μm inner diameter, Dionex) for pre-concentration and desalting before being resolved by nano-reverse phase C18 PepMap analytical column (150 × 75 μm inner diameter, Dionex). Separation was achieved by forming a gradient of 5–85% solvent B (solvent A: acetonitrile/water, 5:95 (v/v), 0.1% formic acid (v/v); solvent B: acetonitrile/water, 95:5 (v/v), 0.1% formic acid (v/v)) over 100 min. The flow rate was maintained at 200 nl/min and electrosprayed into the mass spectrometer using a Z-spray nanoLC source (Waters). The mass spectrometer was operated in a data-directed acquisition mode. Survey scans were acquired from 350 to 1700 *m/z* in positive ion mode with the MS to MS/MS switching, including precursor ion intensity and charge state. MS/MS spectra were acquired from 50 to 1700 *m/z*, and the collision energy varied according to charge state. The RF lens was adjusted for optimal detection of the low *m/z* reporter ions.

##### Data Analysis

ProteinLynx Global server was used to process MS/MS data to generate peak lists. Peak lists were submitted to MASCOT (Matrix Science, London, UK) and searched against a FASTA format of the mouse NCBI protein database using the following search parameters: 150 ppm peptide tolerance; 0.25-Da MS/MS peptide tolerance; 1 maximum missed cleavage; variable methionine oxidation, and two fixed and one variable modification for the iTRAQ chemistry. Only peptides above a 70% confidence were saved for identification and quantification. All spectra of identified proteins were manually checked to ensure that at least three y- and related b-ions were present. Reporter ion intensities were extracted from the corresponding MS/MS spectra using an in-house script, and reporter ions below an intensity threshold of 20 counts were excluded. Peptides ratios that are blank, 0, or >9999 were also removed. We then manually re-inspected each case to ensure any such value was not from an intrinsic biological effect and that protein identifications were based on accurate assignment of MS/MS fragment ions. Based on these criteria, we found no evidence that a peptide could show an expression value of >9999 (27, 28). The relative amount of a peptide in each sample was calculated by dividing the peak areas at 117.1 by the observed mass at *m/z* 115.1. Peak areas were corrected for overlapping isotopes per the manufacturer's instructions. These ratios were ensembled and plotted on a correlogram. We used a self-imposed 2-fold cutoff for reporting a protein as showing significance. The rigorous cutoff was set because a number of the proteins that satisfy criteria for inclusion were achieved on the basis of individual peptides. Moreover, we saw limited technical variations in the prior iTRAQ studies we have conducted (26, 29). This cutoff is more stringent than the 1.3-fold used by several others (27, 30).

##### Manipulation of Dissected Hippocampus

Dissected hippocampi from NBH and ME7 animals 8, 13, and 21 weeks post-inoculation (*n* = 5 per group) were homogenized in 10 volumes of PBS (containing 0.11% diethyl pyrocarbonate) and split and into 2 equal aliquots, which were stored at −80 °C. Subsequently, total protein and total RNA were extracted from these samples, respectively, allowing for correlative protein and mRNA expression to be performed on the same tissue from a single animal.

##### Hippocampal Protein Extraction and Analysis

In our previous studies, there was no marked difference in the protein expression in NBH animals across disease time points so we used a pool of 8-, 13-, and 21-week NBH samples as control (NBH control, *n* = 15), which we compared with ME7 animals at 8, 13, and 21 weeks post-inoculation (*n* = 5 per group). Protein/chemical samples were combined with an equal volume of lysis buffer (4% SDS, 40 mm HEPES, 200 mm KCl containing a mixture of phosphatase inhibitors and complete protease inhibitor mixture). Samples were incubated for 1 h and then centrifuged at 15,000 rpm for 30 min at 4 °C. The supernatant was collected as the SDS-soluble fraction and the protein content quantified using the protein assay from Bio-Rad. These SDS extracts of total hippocampal proteins samples were subsequently diluted into sample buffer, resolved by PAGE, and transferred to nitrocellulose prior to analysis by Western blotting (31).

##### Western Blotting Procedure

Membranes were blocked in 5% nonfat milk for 1 h at room temperature and then incubated in TBS containing 0.1% Tween 20 and one of the following primary antibodies: anti-cow glial fibrillary acidic protein polyclonal antibody (1:5000; DAKO Laboratories); anti-EAAT-2 polyclonal antibody (1:1000; Sigma); anti-Clusterin mAb (1:1000; Cell Signaling); anti-peroxiredoxin-6 mAb (1:1000; Cell Signaling), and 6H4 anti-PrP mAb (1:5000; Prionics). Membranes were incubated with the primary antibodies overnight at 4 °C. The blots were washed and incubated for 1 h with fluorescently labeled anti-sheep secondary antibody at room temperature. Protein band intensities were analyzed using a LiCor Odyssey infrared detection system (LI-COR Biosciences), following the manufacturer's guidelines, and normalized to total protein loading, as measured by colloidal Coomassie Blue staining by an approach described elsewhere (32).

##### Total RNA Extraction and TaqMan Real Time PCR

Total RNAs were extracted from the homogenized hippocampal tissue that was divided into aliquots for protein and transcript analysis as described above. Aliquots of these homogenates were extracted with RNeasy (Qiagen). After treatment with RNase-free DNase (Promega), the concentration of total RNA purified for each sample was measured using a NanoDrop spectrophotometer. 400 ng of total RNA was reverse-transcribed into cDNA in a 20-μl final volume using the iScript reverse transcriptase kit (Bio-Rad). The optimal PCR conditions for each transcript investigated were experimentally determined using the temperature gradient function of the DNA Engine Opticon 2 (MJ Research, Waltham, MA) (data not shown). Reactions were performed in the DNA Engine Opticon 2 real time PCR detection system (MJ Research), and real time PCR efficiencies were calculated from the given slopes in the Opticon 2 Monitor software (MJ Research). Preliminary experiments ensured amplifications were analyzed in the linear range. The following primer and probe sequences were designed with Primer Express software (Applied Biosystems): EAAT-2 (primer sequence 5′-CCC AGC GCC GGG CTG GTC-3′, 5′-GGA CTG CGT CTT GGT CAT TTC G-3′, and 5′Fam-GCT GTG GGC CTG CCA ACG-3′Tamra probe); peroxiredoxin-6 (primer sequence 5′-CCC GGA GGG TTG CTT CTC-3′, 5′ CGT GGT GGC AGG GTA GAG GA-3′, and 5′Fam-CCA GTG TGC ACC ACA GAA C-3′Tamra probe); PrP (primer sequence 5′-AGT CCA ATT TAG GAG AGC CAA GC-3′, 5′-TCA GTC CAC ATA GTC ACA AAG AGG-3′, and 5′Fam-AGC AGC CAG TAG CCA AGG TTC GCC-3′Tamra probe); GFAP (primer sequence 5′-TTT CTC CAA CCT CCA GAT CC-3′, 5′-CCG CAT CTC CAC AGT CTT TA-3′, and 5′Fam-CCA GCC TGG ACA CCA AAT CCG-3′Tamra probe), and Clusterin (primer sequence 5′-AGG AGC TGA ACG ACT CGC T-3′, 5′-GCT TTT CCT GCG GTA TTC C-3′, and 5′Fam-CAG AGC AGT ACA AGG AGC TG-3′Tamra probe). mRNA expression was quantified using a TaqMan RT-PCR kit (Applied Biosystems). EAAT-2, peroxiredoxin-6, PrP, GFAP, and Clusterin mRNA expression in the different samples were normalized for GAPDH gene expression levels with the rodent GAPDH reagents kit (Applied Biosystems), specifically for standardizing gene expression levels to a housekeeping gene, and the results were expressed as relative fold increase or decrease between treatments (ME7/NBH).

##### Mouse Clusterin ELISA

The concentration of Clusterin in plasma from 21-week NBH and ME7 animals was assayed using a commercial mouse quantitative ELISA kit (mouse Clusterin Quantikine ELISA kit, R&D Systems). All reagents, standard dilutions, and samples were prepared as directed in the manufacturer's instructions. Briefly, sera were diluted 1:2000 with calibrator diluent buffer and then added to the ELISA plate in duplicate and incubated for 2 h at room temperature on a horizontal orbital microplate shaker set at 500 rpm. After incubation with the mouse Clusterin conjugate, the reaction was stopped and analyzed spectrophotometrically at 450 nm. The concentration of Clusterin was determined relative to a standard curve generated with recombinant Clusterin. A positive control sample was provided with the kit, which had an assay range of 0.781–50 ng/ml.

##### Histology and Immunohistochemistry

Immunohistochemistry analysis was performed on (10 μm) paraffin-embedded tissue sections from NBH and ME7 animals using antibodies directed against (PrP, synaptophysin, GFAP, Clusterin, EAAT-2, and Prdx6) with previously described protocols (22, 24). In particular, histological detection of PrP^Sc^ was achieved by taking coronal sections from NBH and ME7 animals and immunostaining with anti-PrP mAb 6H4 following formic acid and autoclaving treatment to remove PrP^c^ leaving only PrP^Sc^ species (22, 24). Specific binding was detected using biotinylated secondary antibodies, avidin-biotin-horseradish peroxidase complex kit, and 3,3′-diaminobenzidine as a substrate (Vector Laboratories) to visualize antibody localization according to the manufacturer's instructions. Nuclei were counterstained with hematoxylin. Following double antibody staining of some sections, fluorescent anti-mouse, -rabbit, or -goat secondary antibodies were used to visualize the proteins. Co-localization was demonstrated with the co-localization finder plugin of the ImageJ processing package (National Institutes of Health, Bethesda, MD).

##### Statistical Analysis

All statistical analyses were made using Graph Pad Prism 4.0 (Graph Pad Software Inc., San Diego). A one-way analysis of variance test was applied to biochemical data. A Student's *t* test together with Welch's correction was used to compare mRNA expression data and Clusterin levels.

## RESULTS

### 

#### 

##### Protein Profiling, iTRAQ, and Mass Spectrometry

**FIGURE 1.**
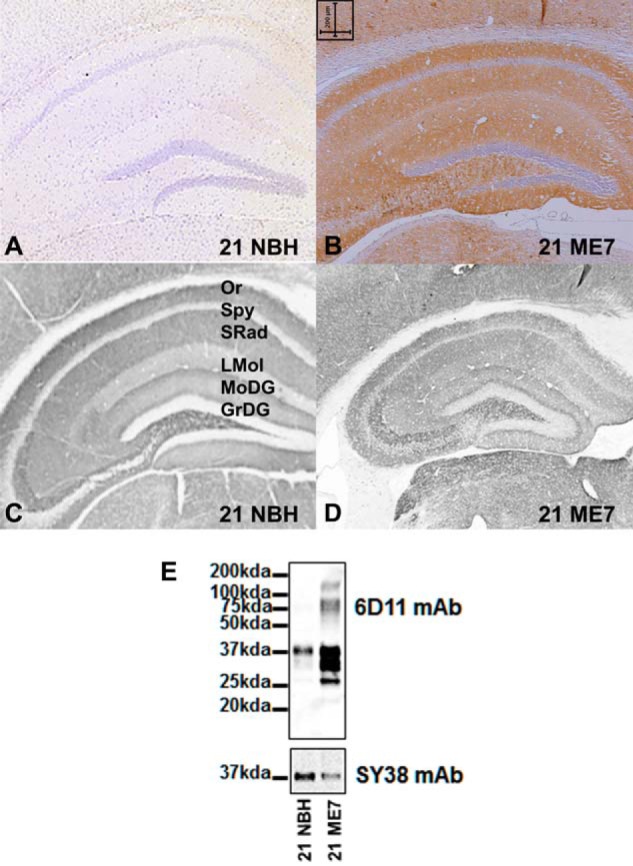
**Basic synaptic architecture in the ME7 model.** Representative immunohistological staining using 6H4 PrP monoclonal antibody on autoclaved formic acid-treated coronal sections from NBH control animals (*A*) are compared with ME7 animals (*B*, showing accumulated misfolded prion (PrP^Sc^; *brown stain*)). Synaptophysin monoclonal antibody (mAb SY38) staining of normal hippocampal strata in NBH animals (*C*) are compared with ME7 animals (*D*, showing synaptic disorganization and neurodegeneration in late stage prion hippocampal tissue). Labeling of hippocampal layers is as follows: *Or*, oriens layer; *Rad*, stratum radiatum; *LMol*, lacunosum molecular layer; *MoDG*, molecular layer dentate gyrus; *GrDG*, granular layer dentate gyrus; *PoDG*, polymorph layer dentate gyrus. *Scale bar*, 200 μm. *E* shows a representative Western blot of total brain homogenates from normal (NBH) or ME7-inoculated mice showing disease-associated increase of PrP (mAb 6H4) and a decrease in synaptic marker protein synaptophysin (mAb SY38) in ME7 compared with NBH animals.

In the ME7 model of prion disease, proteinase K-resistant PrP^Sc^ is readily detected at late-stage disease and is widespread throughout the hippocampus. These features were demonstrated in the cohorts of animals set up for this study and are depicted in the immunocytochemical staining of PrP^Sc^ (Fig. 1, *A* and *B*). In addition, in animals at an advanced stage of disease, the hippocampal formation is clearly reduced in volume, and there is hippocampal cell loss indicated by thinning of the stratum pyramidal of CA1 and a decrease in the intensity and disorganization of staining of several synaptic markers, including the synaptic vesicle protein synaptophysin (Fig. 1, *C* and *D*). Further Western blotting from tissue extracts reveal the disease-induced accumulation of PrP^Sc^ and the robust reduction in synaptic proteins (Fig. 1*E*). Animals displaying this neuronal degeneration show overt changes in a number of affective, cognitive, and motor behaviors and exhibit piloerection and hunched behavior in the home cage (22).

**FIGURE 2.**
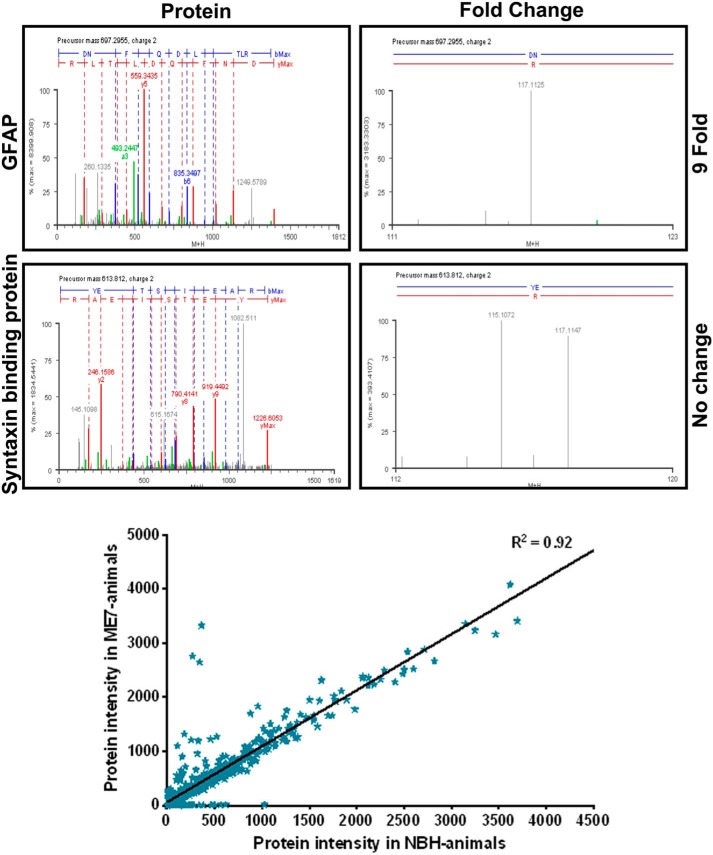
**Representative MS/MS spectra for two peptides identified in this analysis are shown.** *Top* and *middle panels*, 100 μg of homogenate was denatured, reduced/alkylated, trypsin-digested, and labeled with iTRAQ reagents 115.1 and 117.1 in parallel. Both reagents have a reporter group to label primary amines as well as a balance arm. The resulting labeled, complex peptide mixture was mixed and separated by cation exchange chromatography. Following collision-induced dissociation MS/MS analysis of the precursor ion, the reporter groups appear as distinct ions (*m/z* 115–117), and the relative concentration of the peptides is derived from the relative intensities of the reporter ions. MS/MS spectra for Syntaxin-binding protein and GFAP are shown, with the peptide fragmentation and isobaric tag fragmentation that are used to identify (*left panel*) and quantify (*right panel*) protein expression, respectively. All signals with reporter ion intensity of <20 were ignored. This analysis produced robust signals encompassing 2–9 peptides from >200 individual hippocampal proteins. Correlogram showing relative expression of over 200 hippocampal proteins in NBH and ME7 hippocampal extracts. *Bottom panel*, over 200 proteins were predicted using bioinformatics tools. We used a 2-fold change as a cutoff to score changes in protein expression; eight proteins were identified as up-regulated and three proteins as down-regulated in ME7 compared with NBH animals. Four of the up-regulated proteins (GFAP, Clusterin, EAAT-2, and Prdx6) are components of astrocyte.

We pooled hippocampi from five 21-week-old ME7 animals and compared them with age-matched NBH animals. We extracted total protein from membrane homogenates by solubilizing in SDS-containing buffers compatible with affinity labeling with iTRAQ (Applied Biosystems). This involved an upper SDS concentration of 0.1%. We profiled and quantified differences using mass spectrometry. The MS/MS approach causes a fragmentation of the isobaric tags from the parent peptide, and the signals from these are captured at the low molecular weight end of the spectrum. This relative abundance of the signal from the isobaric tags affords quantification of individual proteins from the samples from ME7 and NBH animals, respectively. In the high molecular weight end of the spectrum, the fragmentation of the parent ion provides a signal that is used to identify protein. In the current analysis, NBH animal control samples were labeled with the 115 isobaric tag, and the ME7 animal samples were labeled with the 117 tag. Many peptides fragmented to reveal no change in relative abundance, and this is illustrated in Fig. 2, *middle panel*. Other peptides that were fragmented show clear differences in their relative expression. Among the more striking changes were those associated with several peptides derived from GFAP; the raw signal from one of the peptides for this protein is also shown (Fig. 2, *top panel*).

##### Changes of Peptide Abundance in the Hippocampus of NBH or ME7 Animals

The complete MS/MS spectra collected were first subjected to manual analysis to eliminate spectra in which the NBH- or ME7-related signal was less than 20 counts. The remaining spectra were queried against a mouse database, and the majority of the peptides matched with high MASCOT ion scores (*p* < 0.05). Our MS/MS analysis is based upon quantitative peptide data from >200 individual hippocampal proteins whose expression changes or remains the same (Fig. 2, *bottom graph*). Using a 2-fold change as a cutoff to score changes in protein expression, we found three proteins were down-regulated and eight proteins up-regulated in ME7 animals relative to NBH animals (Table 1). The rigorous cutoff was set because a number of the proteins that satisfy criteria for inclusion were achieved on the basis of single peptides. The displayed proteins did not include synaptophysin, which we previously showed had reduced expression by quantitative immunoblotting (Fig. 1*E*). This supports the notion that the current analysis favored the detection of proteins that were relatively abundantly expressed in the extracted hippocampal proteome. Brief descriptions of the function of the differentially regulated proteins are presented in Table 2. Further information about the identified proteins, which did not change or showed potential differential regulation below our criteria, are listed in Gray (33).

**TABLE 1:** **Proteins differentially expressed in NBH and ME7 hippocampal extracts** Selected up-regulated proteins in ME7 are compared with NBH samples. Only proteins with ratios at least 2-fold higher or lower than the average value (0.9) were considered. Candidate proteins are reported with their associated accession number (in italics below). Eight peptides were up-regulated in ME7 compared with NBH. Selected down-regulated proteins in ME7 are compared to NBH samples. Three peptides down-regulated in ME7 compared with NBH animals are listed.

Up-regulated proteins	-Fold change	Peptides	emPAI score	Down-regulated proteins	-Fold change	Peptides	emPAI score
Cytokine-like-1 *EF108311*	4.3	MAEVDTLK	21.53	*S*-Acetyltransferase *NM145614*	30.5	ILVPEGTR	27.37
Clusterin *NM013492*	2.8	ASGIIDTLFQDR	38.73			GLETIASDVVSLASK	27.37
GFAP *X02801*	3.8	ALAAELNQLR	63.94	Synaptotagmin 1 or 5 *NM009306*	22.9	LTVVILEAK	44.02
		DNFAQDLGTR	125.90			TLVMAVYDFR	59.12
		ESASYQEALAR	64.51			VPTAGK	28.69
		FADLTDAASR	155.76	Ubiquitin-conjugating enzyme *U82627*	13.6	VILQELR	25.38
		IYEEER	64.51				
		LDQLTANSAR	125.90				
		LEAPDADELPR	176.95				
		LQDETNLR	155.76				
Lectin, mannose binding-2 *NM025828*	3.2	DNVDDPTGNFR	20.93				
MAP-1A *AF182208-12*	8.4	AVLDALLEGK	23.89				
		EIQGLFEEK	59.85				
Nesprin-2 *NM001005510*	10.4	MLQQKSR	20.24				
		LQLETMNQK	20.24				
Peroxiredoxin-6 *NM174643*	2.4	NFDEILR	26.99				
		SVDEIR	23.79				
EAAT-2 *AB007810*	35.0	MQEDIEMTK	57.81				
		SELDTIDSQHR	24.29				

**TABLE 2:** **Function of proteins differentially expressed in NBH and ME7 hippocampal extracts** Proteins are reported with their associated accession number (in italic below).

	Function
**Up-regulated proteins**	
Cytokine-like-1 (Cytl1) *EF108311*	Candidate cytokine with unknown function that was originally identified in bone marrow-derived CD34-positive cells but was predominantly expressed in chondrocytes and cartilage (106).
Clusterin (Clu) *NM013492*	Expressed in a variety of tissues where it binds to cells, membranes, and hydrophobic proteins. It is associated with apoptosis and is up-regulated in osteoarthritis and recently highlighted in GWAS for Alzheimer disease (35).
GFAP *X02801*	GFAP is a class-III intermediate filament (37).
Lectin, mannose binding-2 *NM025828*	Recognizes glycosylphosphatidylinositol anchors or sugar residues of glycoproteins and glycolipids and may be involved in the sorting or recycling of proteins and lipids and endoplasmic reticulum-to-Golgi transport of selected proteins (107).
Microtubule-associated protein 1A (MAP-1A) *AF182208-12*	Structural protein involved in the filamentous cross- bridging between microtubules and other skeletal elements (108).
Nesprin-2 *NM001005510*	Maintenance of nuclear organization and structural integrity. Connects nuclei to the cytoskeleton by interacting with the nuclear envelope and with F-actin in the cytoplasm (109).
Peroxiredoxin-6 (Prdx6) *NM174643*	It may play a role in the regulation of phospholipid turnover as well as redox regulation of the cell (34).
EAAT-2 *AB007810*	Solute carrier family 1 (glial high affinity glutamate transporter), member 2; it transports l-glutamate and also l- and d-aspartate. Acts as a symport by co-transporting sodium (36).

**Down-regulated proteins**	
*S*-Acetyltransferase *NM145614*	Component of the pyruvate dehydrogenase complex catalyzes the overall conversion of pyruvate to acetyl-CoA and CO_2_ (110).
Synaptotagmin 1 or 5 *NM009306*	Ca^2+^-dependent synaptic vesicle-trafficking protein, involved in regulation of glial glutamate release (111, 112).
Ubiquitin-conjugating enzyme *U82627*	Regulation of sumoylation with the help of E3 ligases like RANBP2 or CBX4 (63).

##### Astrocytic Response in ME7 Model

**FIGURE 3.**
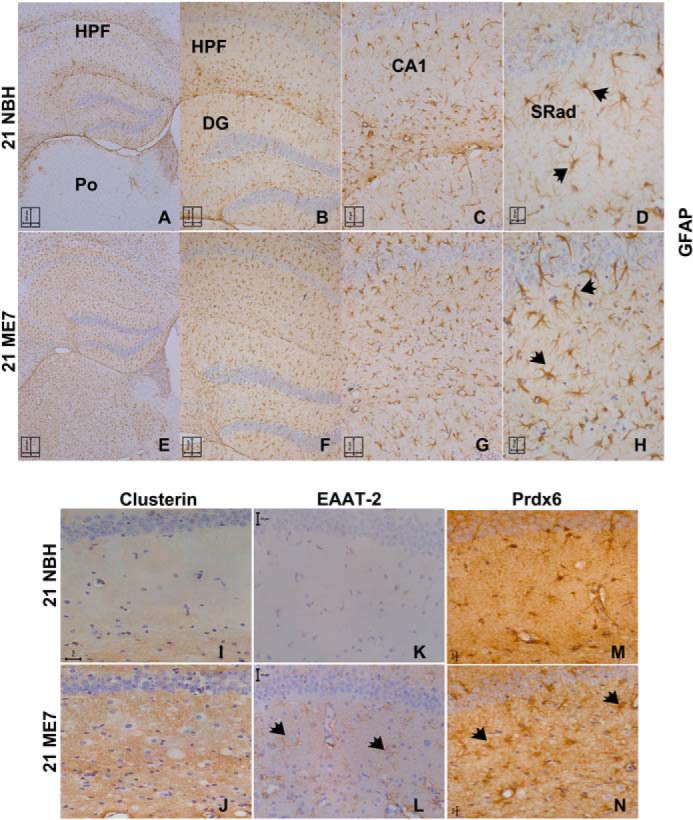
**Reactive astrocytes in the hippocampus of ME7 animals.** Photomicrographs illustrate GFAP expression in NBH and ME7 animals at 21 weeks. Nuclei were counterstained with hematoxylin. GFAP immunoreactivity in astrocytes in the hippocampal layers of NBH animals (see *arrowheads* in *A–D*, ×5, ×10, ×20, and ×40 magnifications, respectively) is shown. At this stage, there is minimal GFAP staining in the cortical regions of NBH animals (data not shown). ME7 animals were strongly positive for GFAP, particularly within the swollen astrocytic processes (see *arrowheads* in *E–H*, ×5, ×10, ×20, and ×40 magnifications, respectively), and at this stage of the disease, there is strong GFAP staining in the cortex (data not shown). Labeling of hippocampal areas: hippocampal formation (*HPF*); dentate gyrus (*DG*); posterior thalamic nucleus (*Po*); cornu ammonis area 1 (*CA1*); stratum radiatum (*SRad*). Brain sections were stained with antibodies against the other astrocyte-associated proteins highlighted in the proteomic analysis, and representative images are shown of the stratum radiatum in NBH and ME7 animals immunostained for Clusterin (*I* and *J*), EAAT-2 (*K* and *L*), and Prdx6 (*M* and *N*) (*arrowheads* are directed to examples of immunoreactive astrocytes). *Scale bar*, 20 μm.

**FIGURE 4.**
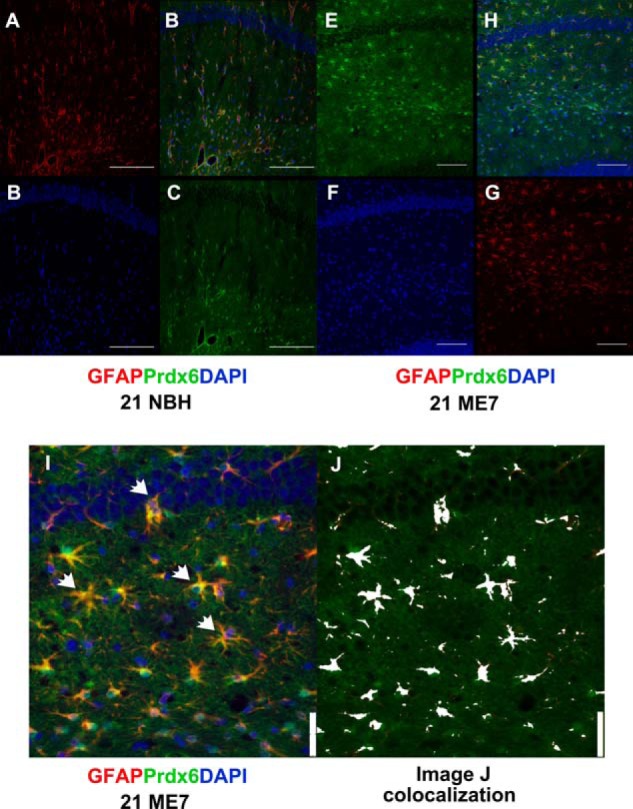
**Prdx6 staining of hippocampus in NBH and ME7 animals.** Representative fluorescent images to illustrate Prdx6 expression in astrocytes in the CA1 hippocampal region of NBH and ME7 animals. *A–D*, co-staining of Prdx6 with GFAP in NBH animals. *A*, GFAP, Texas red; *B*, nuclei, DAPI blue; *C*, Prdx6, FITC green; and *D*, merged image. *Scale bar*, 100 μm. *E–H*, ME7 animals. *E*, Prdx6, FITC green; *F*, nuclei, DAPI blue; *G*, GFAP, Texas red; and *H*, merged image. *Scale bar*, 75 μm. *I*, representative image showing CA1 hippocampal region of high expression coincidence of Prdx6 and GFAP (*arrowheads*) in ME7 animals are shown at a higher magnification. ImageJ co-localization is shown in *white* in the *far right*, illustrating the pixels having both significant *red* and *green signal* (*J*). *Scale bar*, 50 μm.

GFAP was among the most robustly up-regulated protein based on the relative intensity of a number of peptides independently identified. Furthermore, the intensity of the MS signal in NBH samples also indicated that the protein is among the more abundant proteins profiled even in control samples. Immunocytochemical staining for GFAP in hippocampal sections from NBH and ME7 animals shows the abundance of astrocytes in both cohorts and a markedly increased staining in the ME7 samples (Fig. 3, *A–D*, compared with *E–H*). Three of the other candidate proteins that we observed to be up-regulated in ME7 animals included Clusterin, EAAT-2, and Prdx6, which are all known to be predominantly expressed in astrocytes (Fig. 3, *I–N*) (34–36). Immunoreactivity for these molecules is also observed in astrocytes outside the hippocampus (data not shown). We used double immunocytochemistry of Prdx6 and GFAP to confirm whether this protein is expressed and induced in astrocytes or other cells in ME7 animals. Our data revealed co-localization of Prdx6 and GFAP albeit with weaker immunoreactivity of Prdx6 than GFAP; Prdx6 was expressed in NBH tissue (Fig. 4, *A–D*) but was markedly increased in the ME7 animals (Fig. 4, *E–J*). No cells other than astrocytes appear to express Prdx6. ImageJ co-localization is shown in *white* at the far right, illustrating the pixels having both significant *red* and *green* signals (Fig. 4, *I–J*).

##### Estimating Proliferated Changes in ME7

**FIGURE 5.**
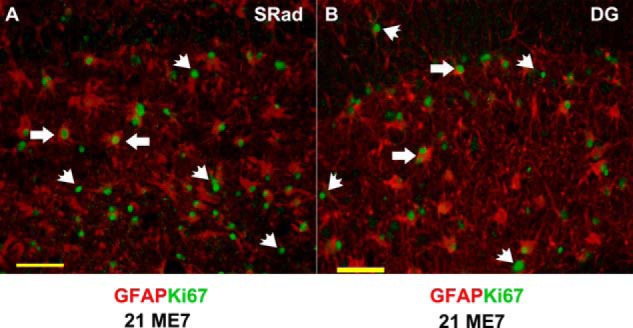
**Expression of the proliferation marker Ki67 in the hippocampus of ME7 animals and its co-localization with astrocyte marker GFAP.** Representative images are from coronal sections showing hippocampal region stratum radiatum (*SRad*) (*A*) and dentate gyrus (*DG*) of ME7 animals stained for Ki67^+^ cells, FITC green and GFAP, Texas red (*B*). Majority of Ki67 staining (FITC green) was restricted to the nucleus (*arrowheads*) and largely distinct from the GFAP (Texas red). A few Ki67^+^ cells were apposed to GFAP (*arrows*), but this was associated with rather than within the GFAP-positive cells. *Scale bar*, 100 μm.

The increased expression of the individual proteins could simply reflect an increase in astrocyte number as a consequence of disease. There is evidence showing that GFAP content is increased in astrocytes during reactive gliosis (37, 38) and also evidence astrocytes may proliferate in some disease states (39). To address this issue, we used Ki67 as a marker of cell proliferation and GFAP double labeling to investigate if there was proliferation of astrocytes during ME7 disease. Using this approach, we observed that in NBH animals Ki67 immunoreactivity is low (data not shown) relative to ME7 animals (Fig. 5). Although there was increased Ki67 in ME7 animals, we did not observe significant co-localization of Ki67 and GFAP (Fig. 5, *arrowheads*). In instances of Ki67 and GFAP apposition, this staining appeared in associated cells (Fig. 5, *arrows*). This is consistent with our recent report in which we demonstrate that the microglial population was the main cell type proliferating during prion disease (21). This suggests that the increased level of the astrocytic protein in the hippocampus of ME7 animals is not determined by their proliferation (Fig. 5).

##### Cellular Regulation of Glial Protein Expression in ME7 Disease

**FIGURE 6.**
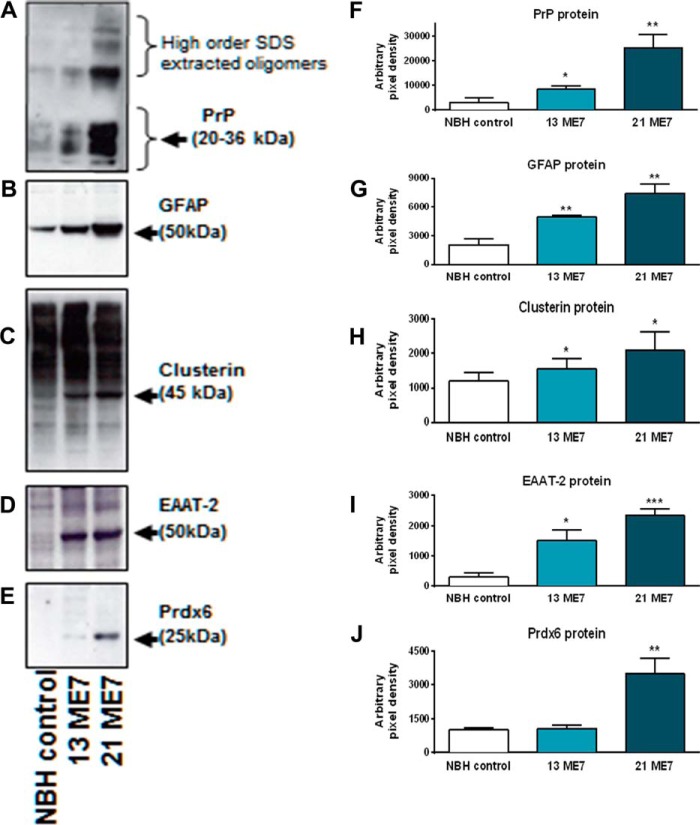
**Western blot analysis to verify the protein changes observed from MS analysis.** Quantitative Western blotting of astrocytic proteins in hippocampal homogenates (brain equivalent, 20 μg) from normal animals (pooled NBH controls) compared with ME7 animals (13 and 21 weeks) Samples were probed for the presence of PrP (mAb 6H4) and candidate up-regulated proteins (GFAP, EAAT-2, Clusterin, and Prdx6). *A–E* shows representative experimental blots. *1st lanes*, NBH control; *2nd lanes*, 13-week ME7; and *3rd lanes*, 21-week ME7. *F–J*, densitometric data in each *bar* represents means ± S.E. from *n* = 5 animals. The *error bar* represents direct comparison between the protein expression in NBH control samples and the ME7 animals. * signifies *p* < 0.05; ** signifies *p* < 0.01; *** signifies *p* < 0.001.

We investigated progressive changes in the identified glial genes and their expressed proteins across disease. Five hippocampi from ME7 and NBH animals at 8, 13, and 21 weeks were homogenized as individual samples and divided in 2 aliquots from which either protein or total RNA was extracted. For quantitative Western blot analyses, we used a pool of SDS buffer-extracted 8-, 13-, and 21-week NBH hippocampal homogenates as control (NBH control), which was compared with 13- and 21-week ME7 animals and probed with antibodies against PrP, GFAP, Clusterin, EAAT-2, and Prdx6 (Fig. 6, *A–J*). The presence of an increasing level of PrP^c^/PrP^Sc^ in each sample was indicated by the increasing anti-PrP antibody immunoreactivity and the emergence of high order oligomers that resist dissociation by SDS at both the middle and late stages of the disease (Fig. 6, *A* and *F*, *p* < 0.01) (32). GFAP immunoreactivity increased with the disease (Fig. 6*B*), and quantification showed a significant increase relative to NBH control at the 13-week time point (Fig. 6*B*, *p* < 0.01) that further increased by 21 weeks (Fig. 6*B*, *p* < 0.01).

Immunoreactivity for Clusterin (Fig. 6*C*) is smeared in keeping with previous data for this highly glycosylated protein (40). Nevertheless, we observed a progressive increase in the immunoreactivity for Clusterin, which resolves as an apparent unglycosylated band (*arrow* in Fig. 6*C*) and the various glycosylated states (*smear* in Fig. 6*C*) that progressively increase at 13 and 21 weeks. We quantified the intensity of the unglycosylated/glycosylated protein band at 45 kDa and showed that this increase was significant relative to NBH control at both 13 and 21 weeks (*p* < 0.01). Similarly, EAAT-2 showed increased expression at 13 weeks that progressed further at 21 weeks (Fig. 6*D*), and quantification of these blots showed that EAAT-2 expression is significantly increased at 13 weeks and progresses to an elevated level at 21 weeks (Fig. 6*I, p* < 0.05 and *p* < 0.001, respectively). Prdx6 expression was also significantly elevated at the late stage of the disease (Fig. 6, *E* and *J*, *p* < 0.01) consistent with the independent identification of its induction in the iTRAQ analysis and by immunohistochemistry (Fig. 3, *L* and *N*). In contrast, this protein does not seem to be significantly elevated at the earlier time point; thus, unlike GFAP and EAAT-2, Prdx6 is only significantly induced later in ME7 disease.

##### Transcriptional Changes in Clusterin, Peroxiredoxin-6, PrP, GFAP, and EAAT-2

**FIGURE 7.**
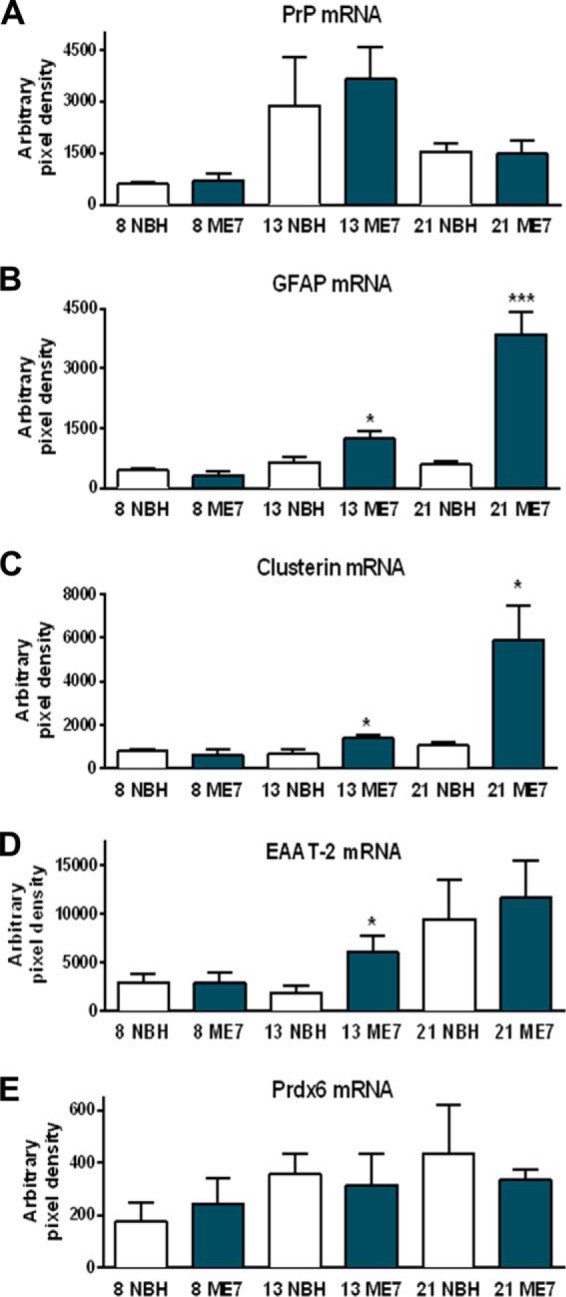
**TaqMan RT-PCR.** TaqMan RT-PCR analysis of EAAT-2, Prdx6, PrP, GFAP, and Clusterin mRNAs in NBH is compared with ME7 animals. *A*, PrP mRNA appears unchanged, although the levels were higher at the mid-stage of the disease. *B* and *C*, Clusterin and GFAP transcription were increased ∼5-fold (*, *p* < 0.05; ***, *p* < 0.001, respectively), compared with NBH at the late stage of the disease. *D*, there was no difference in the levels of EAAT-2 transcript at the late stage of the disease, although there was a significant increase in EAAT-2 mRNA at the mid-stage of disease, compared with NBH. *E*, there was no transcriptional regulation of Prdx6 associated with the disease propagation.

Previous studies indicate that the increased expression of GFAP is directly related to a transcriptional response. However, the differential increased expression in PrP, Clusterin, and Prdx6 identified in this study indicates that there may not be simple relationship between transcription and protein expression. TaqMan RT-PCR was performed to quantify the expression of the mRNA species. The expected PCR product size for each gene product was initially confirmed by agarose gel electrophoresis, and additionally the no-template controls showed no amplification (data not shown). The RT-PCR analysis compared changes in PrP, GFAP, Clusterin, EAAT-2, and Prdx6 mRNAs of ME7 and NBH animals (Fig. 7, *A–E*). It should be noted that these mRNAs are taken from the same tissue used to measure protein expression levels. Despite the large increase in PrP protein, there is no differential change in the PrP mRNA between NBH and ME7 animals (Fig. 7*A*). We observed that in ME7 animals at the late stage of the disease, GFAP and Clusterin transcription was increased ∼5-fold (Fig. 7, *B* and *C*, *p* < 0.001 and *p* < 0.05, respectively), compared with NBH. There was no difference in the levels of EAAT-2 transcript at the late stage of the disease between ME7 and NBH animals, although there was a significant increase in EAAT-2 mRNA at the middle stage of disease, compared with NBH (Fig. 7*D*). The data suggest that this is due to low level of expression in the NBH group at this time point. Expression values for Prdx6 mRNA were considerably lower than those of the other genes (Fig. 7*E*) and did not increase with disease progression. Increased Prdx6 protein expression is not supported by transcriptional up-regulation of Prdx6 mRNA.

##### Does Glia-produced Clusterin Appear in Blood as a Potential Biomarker?

**FIGURE 8.**
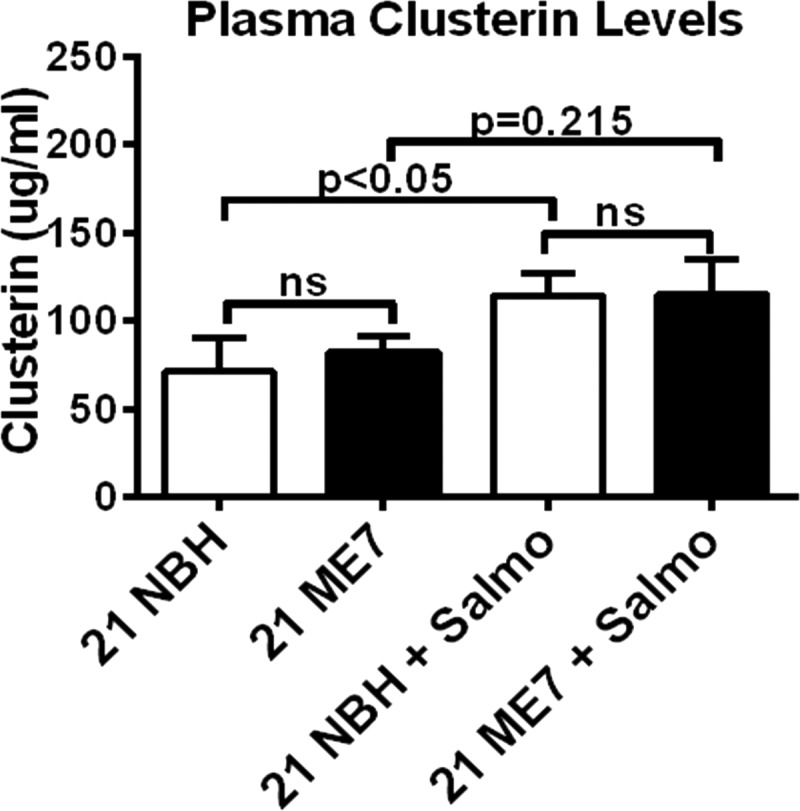
**Measurement of Clusterin in serum.** The levels of Clusterin were measured by quantitative sandwich ELISA in serum of NBH and ME7 animals with or without systemic *S. typhimurium* infection. S.E. values are shown, and there were no statistical (*ns*) differences between NBH and ME7 animals in both groups.

We have previously shown that a number of typical acute phase proteins serum amyloid A, complement C3, pentraxin 3, and α2-antiplasmin are induced in the brain of animals with prion disease, but there is no induction of message in the liver despite the presence of systemic deposition of PrP^Sc^ (19). The induction of the acute phase protein Clusterin in astrocytes is of particular interest because it has been suggested that this protein may be a useful serum biomarker in patients with AD (41). Thus, using a commercially available sandwich immunoassay, we examined the utility of Clusterin as a candidate serum biomarker for prion disease. We measured its concentration in samples from NBH and ME7 animals, and we showed that serum levels of Clusterin were unchanged (Fig. 8*A*, 72.02 ± 9.5 and 83.17 ± 4.5 μg/ml). Despite the significant increase of this protein in the hippocampus, cortex, and thalamic brain regions of animals with prion disease, the protein does not leave the brain in sufficient amounts to be detected in serum. We also examined whether systemic infection with *Salmonella typhimurium* will alter the levels of Clusterin (42). Overall infection with *Salmonella* increased the levels of circulating Clusterin in NBH and ME7 animals by 40% (*p* < 0.05) and 35% (*p* = 0.215), respectively, compared with uninfected animals, but there was no difference between NBH *Salmonella* and ME7 *Salmonella* animals (Fig. 8*A*, 112.1 ± 14.3 and 112.4 ± 21.6 μg/ml).

## DISCUSSION

The ME7 model of prion disease has generated increasingly detailed insights into the evolution of the pathology of this neurodegenerative disease and underlying mechanisms (21, 22, 43–45). In this study, we have investigated the hippocampal proteome at late-stage prion disease. This study uses the iTRAQ technique to simultaneously identify and quantify the hippocampal proteome in animals with ME7- induced prion disease (25, 26, 46). This analysis produced robust signals encompassing 2–9 peptides from >200 individual hippocampal proteins. Using a 2-fold change as a cutoff to score changes in protein expression, we identified eight proteins as up-regulated and three proteins as down-regulated in ME7 animals.

We specifically focused on four of the up-regulated proteins (GFAP, Clusterin, EAAT-2, and Prdx6), which are known to be expressed by astrocytes (47, 48). GFAP up-regulation is a dominant feature of the proteome of prion-diseased brain tissue (32, 49, 50), and the other three proteins all independently associate with astrocytes and show differential increases in expression as the prion disease progresses to end stage. There is a steady increase of GFAP, Clusterin, and EAAT-2 throughout the disease time course with a more restricted onset of increased expression exhibited by Prdx6. The regulation of glial expressed molecules is also distinct when considering the comparison of protein and mRNA expression. These analyses were made by Western blotting and transcriptionally measuring gene expression in protein and mRNA extracted from the same tissue. This shows that the increase in certain glial proteins is matched by transcriptional regulation, whereas in the case of Prdx6, the protein increase seems independent of a transcriptional response. Some of these changes described above are supported by observations in microarray time course studies from other prion strains (50, 51).

The observation that Ki67 staining does not coincide with disease progression shows that the changes in protein expression we have investigated are taking place within a numerically constant population of astrocytes. We observed that Ki67 labeling of astrocytes was negligible, which contrasts with microglia that have a pronounced proliferative response (21). Previous investigations of the microglial response have shown that they contribute to the pathogenesis of prion disease (24, 52). Selective inhibition of microglia proliferation slowed disease progression (21). However, less is known about the roles played by the astroglia.

*In vitro* studies have shown that both neurons and astrocytes are capable of sustaining efficient prion propagation independently, leading to the production of PrP^Sc^ (53), but the kinetics of their production in resting and activated glia have not been studied. Neuronal synthesis of PrP^c^ has been shown to be important, as reversal of prion disease is possible through selective reduction of neuronal PrP^c^ in mice with established prion infection (54). However, there is abundant evidence that non-cell autonomous influences are intimately involved in disease pathogenesis. PrP^c^ expression in astrocytes is critical for sustaining cell-to-cell interactions, neuronal differentiation, and survival (55), but astrocytes accumulate prion aggregates leading to reactive astrocytosis (56) that spares astrocytes with bystander effects on PrP^c^-null neurons (57).

Astrocyte-specific expression of hamster prion protein renders PrP knock-out mice susceptible to prion disease despite the lack of neuronal PrP^c^ (15). Others confirmed that PrP expression on neurons or astrocytes was sufficient for prion-induced neurodegeneration (58), but PrP^c^ expression on follicular dendritic cells is also important for prion replication (59, 60). Equally important in disease pathogenesis is the role of neuron-glia cross-talk likely initiated by an interaction between microglia-secreted molecules and astrocytes, which then impact on neurons (61, 62). This highlights glial cells playing a crucial part in disease as has been observed in several other neurodegenerative conditions (16, 64).

The proteins identified in the proteomic screen exhibit functions that support a potential role as modulatory determinants of disease progression. Although GFAP is essentially a structural protein, its induction in astrocytes underpins important morphological changes that the astrocytes use to extend their sphere of influence and facilitate cellular structures and function by determining the shape and controlling the movement of these cells (65). In the case of Clusterin, several associated activities suggest it regulates neurodegeneration (66, 67). Clusterin functions include extracellular protein chaperoning, lipid carriage, and participation in stress responses, all of which are neuroprotective. In the context of prion disease, Clusterin shows an ability to bind misfolded protein with high avidity (68), and it has been shown to reduce the cytotoxicity of amyloid-β in AD (69). However, Clusterin is also known to collaborate with the complement cascade in both the CNS and the periphery, an interaction that may help with misfolded protein clearance (70, 71). The role played by these pathways and their potential convergence with each other has received a renewed interest following the observation that Clusterin and the C1q receptor, an early component of the complement cascade, are significant genetic determinants of late onset AD (72, 73).

Clusterin was found to be increased in plasma of AD patients, and the levels were reported to be associated with severity and progression of disease (41, 74, 75), but others disagree (76–78). We examined whether changes in brain concentrations of Clusterin in ME7 animals was reflected in serum. We tested sera from 21-week NBH and ME7 animals at a time point when there was robust neurodegeneration and peripheral deposition of PrP^Sc^. Crucially, we could be certain that the animals had not yet progressed to terminal disease, when urinary incontinence could cause systemic inflammation sufficient to trigger hepatic acute phase response independent of the ensuing neurodegeneration. Our results showed no difference in serum levels of Clusterin between NBH and ME7 animals. These data in which peripheral confounds have been well controlled caution against the idea that raised brain levels of acute phase proteins or centrally produced components of innate immune cells can be detected in serum.

There was a significant difference in serum Clusterin levels between control and animals infected with *S. typhimurium*, but there was no difference between cohorts of *S. typhimurium-*infected NBH and ME7 animals (42). This shows that serum Clusterin estimates are unsuitable as a biomarker for prion disease. Protein and mRNA levels of pro-inflammatory cytokines like IL-1, IL-6, and TNF-α are elevated in the brain, peripheral lymphoid tissue, and serum of terminal prion animals (19, 79, 80). The macrophages, monocytes, and astrocytes responsible for these signals can induce expression of acute phase proteins like Clusterin (81, 82). Clusterin levels are elevated in synovial fluid of rheumatoid arthritis and osteoarthritis patients (83, 84) and in the cerebrospinal fluid of patients with AD, Parkinson disease, and multiple sclerosis (85–87), as well as in the urine of patients with kidney injury or bladder cancer (88, 89). It cannot be ruled out that an elevated level of Clusterin in the plasma of AD patients reflects systemic disease (90). The consensus is that although acute phase proteins are highly sensitive indicators of inflammation and tissue injury, they lack specificity (91, 92).

The induction of EAAT-2 and Prdx6 represents a coordinated increase in molecules that provide a neuroprotective role. EAAT-2 is responsible for the majority of steady state glutamate uptake in the brain (93), and the increased expression of this molecule will buffer against the neurotoxic effect of elevated extracellular levels of this transmitter (94). In other protein misfolding diseases, there is a clear precedent for a targeted disruption in EAAT-2 function (95); however, the significance of glutamate toxicity in prion disease may be more limited but is less well understood. Evidence from a recent transcript analysis of prion disease animals also suggests that excitotoxic signaling may contribute to the ongoing disease process (96). This would mean that EAAT-2 expression reflects an important secondary effect to buffer glutamate that arises from the progressive primary neurodegeneration. In a similar way, Prdx6 belongs to a family of 25-kDa peroxidases with a single redox-active cysteine thought to function as antioxidants (97, 98). Although Prdx6 has a reported phospholipase A_2_ activity its role in the glial response is likely to be as an antioxidant (99). Our analysis of this protein and its mRNA, while supporting its appearance in disease glial expression, suggests that it is regulated at the level of protein.

We specifically focused on the astrocyte-related proteins in this report, but the remaining up-regulated proteins are also of interest and have been briefly described in Table 2. In particular, their potential role in mediating changes in innate immune regulation and neuronal structural remodeling also suggests the existence of possible new targets for therapeutic intervention. Other prions strains (22L and 79a) exhibit some overlapping behavioral deficits and neuropathology with ME7 (100, 101), but we have limited evidence that our biochemical observations in ME7 prion disease are the same as in other strains. A more detailed discussion of potential strain differences that have not been investigated is beyond the scope of this report. However, there is evidence that the proteins we have investigated are also differentially regulated in other prion diseases (32, 49–51, 102–105). The current analysis, although restricted to identifying and characterizing the most abundant components of the degenerating hippocampal proteome, suggests a complex, multifaceted glial response capable of a homeostatic response that supports morphological, metabolic, proteostatic, and neurochemical changes in the chronic degenerating brain.

## Footnotes

2 The abbreviations used are:

AD: Alzheimer disease
PrP^c^: normal cellular PrP
PrP^Sc^: proteinase K resistant form of PrP
GFAP: glial fibrillary acidic protein
NBH: normal brain homogenate
Fam: fluorophore 6-carboxyfluorescein
Tamra: tetramethylrhodamine.
